# Identification and verification of plasma protein biomarkers that accurately identify an ectopic pregnancy

**DOI:** 10.1186/s12014-023-09425-w

**Published:** 2023-09-15

**Authors:** Lynn A. Beer, Xiangfan Yin, Jianyi Ding, Suneeta Senapati, Mary D. Sammel, Kurt T. Barnhart, Qin Liu, David W. Speicher, Aaron R. Goldman

**Affiliations:** 1 Molecular and Cellular Oncogenesis Program, The Wistar Institute, 3601 Spruce Street, Philadelphia, PA 19104 USA; 2 Department of Obstetrics and Gynecology, University of Pennsylvania, Philadelphia, PA USA; 3 Department of Biostatistics and Informatics, Colorado School of Public Health, Aurora, CO USA; 4 Department of Biostatistics, Epidemiology and Informatics, University of Pennsylvania, Philadelphia, PA USA

**Keywords:** Ectopic pregnancy, Miscarriage, Proteomics, Targeted MS, Biomarkers, Biomarker signatures

## Abstract

**Background:**

Differentiating between a normal intrauterine pregnancy (IUP) and abnormal conditions including early pregnancy loss (EPL) or ectopic pregnancy (EP) is a major clinical challenge in early pregnancy. Currently, serial β-human chorionic gonadotropin (β-hCG) and progesterone are the most commonly used plasma biomarkers for evaluating pregnancy prognosis when ultrasound is inconclusive. However, neither biomarker can predict an EP with sufficient and reproducible accuracy. Hence, identification of new plasma biomarkers that can accurately diagnose EP would have great clinical value.

**Methods:**

Plasma was collected from a discovery cohort of 48 consenting women having an IUP, EPL, or EP. Samples were analyzed by liquid chromatography-tandem mass spectrometry (LC-MS/MS) followed by a label-free proteomics analysis to identify significant changes between pregnancy outcomes. A panel of 14 candidate biomarkers were then verified in an independent cohort of 74 women using absolute quantitation by targeted parallel reaction monitoring mass spectrometry (PRM-MS) which provided the capacity to distinguish between closely related protein isoforms. Logistic regression and Lasso feature selection were used to evaluate the performance of individual biomarkers and panels of multiple biomarkers to predict EP.

**Results:**

A total of 1391 proteins were identified in an unbiased plasma proteome discovery. A number of significant changes (FDR ≤ 5%) were identified when comparing EP vs. non-EP (IUP + EPL). Next, 14 candidate biomarkers (ADAM12, CGA, CGB, ISM2, NOTUM, PAEP, PAPPA, PSG1, PSG2, PSG3, PSG9, PSG11, PSG6/9, and PSG8/1) were verified as being significantly different between EP and non-EP in an independent cohort (FDR ≤ 5%). Using logistic regression models, a risk score for EP was calculated for each subject, and four multiple biomarker logistic models were identified that performed similarly and had higher AUCs than models with single predictors.

**Conclusions:**

Overall, four multivariable logistic models were identified that had significantly better prediction of having EP than those logistic models with single biomarkers. Model 4 (NOTUM, PAEP, PAPPA, ADAM12) had the highest AUC (0.987) and accuracy (96%). However, because the models are statistically similar, all markers in the four models and other highly correlated markers should be considered in further validation studies.

**Supplementary Information:**

The online version contains supplementary material available at 10.1186/s12014-023-09425-w.

## Background

Distinguishing a normal intrauterine pregnancy (IUP) from abnormal gestations is a clinical challenge because there is no definitive noninvasive diagnostic test when ultrasound is non-diagnostic. Pregnant women presenting with lower abdominal pain and/or vaginal bleeding will ultimately be diagnosed witha viable IUP, a miscarriage or early pregnancy loss (EPL), or an ectopic pregnancy (EP). EP occurs in 1–2% of pregnant women and is a leading cause of maternal mortality and morbidity, accounting for 6% of pregnancy-related deaths [1, 2], whereas EPL affects 10–20% of pregnancies [3]. Clinical management of these three outcomes is drastically different, and a timely and accurate diagnosis is imperative because undiagnosed EP can be fatal. Currently, serial β-human chorionic gonadotropin (β-hCG, gene name: CGB) and progesterone are the most widely used serum biomarkers for evaluating pregnancy outcome when ultrasound is inconclusive [4–6]. Likewise, serial β-hCG levels and pelvic ultrasound are the standard methods of diagnosing an EP. However, neither method can definitively diagnose an EP with sufficient and reproducible accuracy at very early stages [1, 7].

Proteomics-based discovery of serum/plasma biomarkers of early pregnancy complications as well as other clinical disorders is feasible but challenging because: (1) plasma and serum proteomes are very complex; (2) abundant plasma proteins that are present in the mg/ml range severely limit detection of lower abundance proteins; and (3) most clinical biomarkers are present in the ng/ml to pg/ml range or even lower [8–10]. These challenges have been partially addressed by major advances in liquid chromatography-tandem mass spectrometry (LC-MS/MS) instrumentation and methodologies.

Proteomics discoveries of serum/plasma typically identify moderate numbers (>10) of candidate biomarkers that need to be validated in independent clinical patient cohorts to determine which biomarkers are likely to have sufficient diagnostic accuracy. A major gap exists between candidate biomarker discovery and development of a commercial biomarker assay because, typically, robust, high sensitivity quantitative assays for newly discovered biomarkers are not available and the specificity of non-clinical enzyme-linked immunosorbent assays (ELISAs) are often ambiguous, especially when closely related protein isoforms are present in the sample. Further, in some cases, antibody-based assays have been multiplexed for high-throughput analysis of candidate biomarker panels, such as using the Bio-Plex platform [11, 12]; however, antibodies that recognize specific proteins may not be available. To bridge this gap, we developed a parallel reaction monitoring mass spectrometry (PRM-MS)-based targeted assay that distinguishes between candidate early pregnancy biomarkers with high specificity. Using this assay, we determined the minimum number of protein biomarkers needed for accurate EP diagnosis and demonstrated that this panel of biomarkers can accurately distinguish an EP from other pregnancy outcomes.

## Materials and methods

### Study design

For both the discovery (n = 48; 16 IUP, 16 EPL, and 16 EP) and verification (n = 74; 25 IUP, 24 EPL, and 25 EP) cohorts, plasma samples were collected prospectively from consenting women with symptomatic early-stage pregnancies. For inclusion in this study, participants were required to meet the following criteria: (1) abdominal pain and/or vaginal bleeding; (2) 5–10 weeks of gestation; (3) no chronic medical conditions such as diabetes or obesity; and (4) informed consent to participate in data and sample collection for the Ectopic Pregnancy Biomarkers Bank. Specimens were selected for each cohort from the biobank such that the three pregnancy outcome groups (IUP, EPL, and EP) had similar distributions for gestational age (GA).

GA (based on last menstrual period and/or ultrasound), race, ethnicity, β-hCG, and maternal age were recorded for each subject upon initial examination, if available, and pregnancy outcome was obtained at the time of sample collection or shortly thereafter. Pregnancy outcomes were classified based on consensus definitions for formal diagnosis [13]. Patient characteristics for the discovery and verification cohorts are reported in Table 1.

**Table 1:** Demographic and clinical characteristics of the discovery (N = 48) and verification (N = 74) cohorts

	Discovery Cohort			Verification Cohort		
	**IUP (N = 16)**	**EP (N = 16)**	**EPL (N = 16)**	**IUP (N = 25)**	**EP (N = 25)**	**EPL (N = 24)**
**Race, n (%)**						
Black	13 (81)	15 (94)	7 (44)	15 (60)	16 (64)	12 (50)
White	1 (6)	1 (6)	4 (25)	3 (12)	6 (24)	7 (29)
Other	2 (13)	0 (0)	0 (0)	7 (28)	3 (12)	5 (21)
Unknown	0 (0)	0 (0)	5 (31)	0 (0)	0 (0)	0 (0)
**Ethnicity, n (%)**						
Hispanic	0 (0)	0 (0)	2 (12.5)	5 (20)	3 (12)	1 (4)
Non-Hispanic	14 (87.5)	16 (100)	12 (75)	20 (80)	21 (84)	23 (96)
Unknown	2 (12.5)	0 (0)	2 (12.5)	0 (0)	1 (4)	0 (0)
**Mean hCG (mIU/mL)**	29,983	8,706	24,892	47,150	5,924	21,177
Unknown, n (%)	0 (0)	0 (0)	0 (0)	2 (8)	2 (8)	5 (21)
**Mean maternal age (yrs)**	25	28	22	27	29	32
Unknown, n (%)	0 (0)	0 (0)	10 (62.5)	0 (0)	0 (0)	0 (0)
**Mean GA based on last menstrual period**						
Days	45	48	63	54	46	71
Unknown, n (%)	0 (0)	0 (0)	1 (6)	0 (0)	0 (0)	0 (0)
**Mean GA based on ultrasound**						
Days	44	NA	49	49	NA	52
Unknown, n (%)	0 (0)	0 (0)	1 (6)	2 (8)	0 (0)	3 (13)

### Plasma collection and processing

Blood was collected by venipuncture into K2EDTA plasma tubes (BD, Franklin Lakes, NJ), and centrifuged for 10 min at 1,500 x g at room temperature. Plasma was aliquoted in 500 µl volumes into cryotubes, snap frozen using liquid nitrogen within 2 h of blood collection, and stored at -80 °C. Before downstream processing was performed, samples were thawed briefly in a RT water bath with intermittent periods of cooling on ice to prevent sample warming above 0–4 °C. Thawed samples were centrifuged for 10 min at 12,000 x g at 4 °C, aliquoted into smaller volumes (40–100 µl), snap frozen using liquid nitrogen, and stored at -80 °C until used.

### IGY-14/Supermix depletion

Samples were depleted of abundant plasma proteins using IGY-14 and Supermix immunodepletion columns (Sigma-Aldrich, St. Louis, MO) connected in tandem as previously described [14]. Typically, 100 µl (discovery cohort) or 50 µl (verification cohort) aliquots of plasma were thawed, centrifuged for 10 min at 12,000 x g at 4 °C, diluted five-fold with equilibration buffer, filtered through a 0.22 μm microcentrifuge filter, and injected onto the columns. The flow-through fractions containing unbound proteins were collected, pooled, and concentrated using a 10 K MWCO centrifugal filter unit.

(MilliporeSigma, Burlington, MA), concentrator membranes were extracted with 1% SDS and extracts were combined with the concentrated sample. Concentrated samples were snap frozen, dried using a SpeedVac centrifugeand stored at -20 °C prior to 1-D SDS-PAGE and LC-MS/MS analysis.

### SDS-PAGE/In-gel trypsin digestion

For plasma samples collected from the discovery cohort, IGY-14/Supermix-depleted samples were resuspended in SDS sample buffer, loaded onto pre-cast NUPAGE gels (Thermo Fisher Scientific, Waltham, MA), and separated using MES running buffer (Thermo Fisher Scientific) until the tracking dye had migrated 1.6 cm. Gels were stained with Colloidal Blue (Thermo Fisher Scientific), and the entire gel lane was excised and divided into six fractions, based on gel band staining, as previously described [15]. Fractions were digested overnight using 20 ng/ml modified trypsin. For plasma collected from the verification cohort, samples were processed similarly as described above with the exception that samples were run for 0.5 cm onto gels followed by overnight digestion using 10 ng/ml modified trypsin [15]. Digested samples were dried using a SpeedVac centrifuge and stored at -20 °C. Dried samples were re-suspended in 0.1% formic acid/3% ACN or 0.1% formic acid prior to LC-MS/MS discovery or PRM-MS verification, respectively.

### Stable isotope labeled (SIL) peptide standards preparation

Individual “heavy” SIL peptide stock solutions were prepared as follows: SpikeTides-TQL peptides (JPT Peptide Technologies, Berlin, Germany) were cleaved from their quantification tag (Qtag) prior to stock solution preparation [16]. Briefly, 1 nmol of each dried SIL peptide was solubilized in 25 mM ammonium bicarbonate/20% ACN and digested in-solution with 10 ng/µl trypsin (enzyme/peptide ratio of 1:100) in 25 mM ammonium bicarbonate overnight. Cleaved and digested SIL peptides were dried using a SpeedVac centrifuge, resuspended, and aliquoted in stock solutions of 10 pmol/µl in 10% ACN/2% formic acid. Additionally, dried SpikeTides-L and Maxi SpikeTides-QL peptides (JPT Peptide Technologies) were resuspended and aliquoted in stock solutions of 10 pmol/µl in 10% ACN/2% formic acid, and AQUA peptides (Thermo Fisher Scientific) were aliquoted in stock solutions at 5 pmol/µl in 5% ACN.

Stock solutions of cleaved SpikeTides-TQL (15 total), Maxi SpikeTides-QL (3 total), SpikeTides-L (16 total), and AQUA QuantPro peptides (2 total) were pooled at fmol amounts ranging from ~4 to 70 fmol each based on MS signal intensity that was pre-determined in quality control analyses of individual peptides. The pooled SIL peptide stock solution (10 pmol/µl) to be used for all subsequent quantitation analyses was aliquoted, snap frozen, and stored at -20 °C. Prior to PRM-MS analysis, the pooled SIL peptide stock solution was thawed and diluted ten-fold to a final concentration of 1 pmol/µl in 0.1% formic acid/3% ACN/0.004% PEG. Next, 5 µl (5 pmol) was added to resuspended digests (35 µl) containing the equivalent of 15 µl of original plasma. PRM-MS sample injections (see below) contained the equivalent of 3 µl original plasma and 1 pmol of pooled SIL peptide standards.

### LC-MS/MS

Samples were analyzed on a Q Exactive HF mass spectrometer (Thermo Scientific) equipped with a nanoACQUITY ultrahigh pressure liquid chromatography (UPLC) System (Waters, Milford, MA) and a column heater maintained at 45 °C. Tryptic digests were injected onto a UPLC Symmetry trap column (180 μm i.d. x 2 cm packed with 5 μm C18 resin; Waters), and peptides were separated by reversed phase-ultra high pressure liquid chromatography (RP-UPLC) on a BEH C18 nanocapillary analytical column (75 μm i.d. x 25 cm, 1.7 μm particle size, Waters) at a flow rate of 200 nl/min. Solvent A was Milli-Q (MilliporeSigma) water containing 0.1% formic acid, and solvent B was acetonitrile containing 0.1% formic acid. For the discovery cohort, peptides were eluted using a 70 min LC gradient as previously described [15].

### PRM-MS

For the verification cohort, samples were analyzed on a Q Exactive HF mass spectrometer (Thermo Scientific) equipped with a nanoACQUITY UPLC System (Waters, Milford, MA) as described above. Peptides were eluted at 200 nl/min using an acetonitrile gradient consisting of 5–30% B over 110 min, 30–40% B over 10 min, 40–80% B over 5 min, 80% B for 10 min before returning to 5% B over 0.5 min. The column was re-equilibrated using 5% B at 300 nl/min for 5 min before injecting the next sample. To minimize carryover, a blank was run between each experimental sample by injecting water and using a 30 min gradient with the same solvents. The PRM method consisted of a full MS scan (m/z 375–1150) acquired in profile mode at 30,000 resolution, followed by up to 20 MS/MS scans from an inclusion list containing the m/z, charge state, and retention time ± 5–6 min for each targeted peptide. PRM scans were acquired in profile mode at 30,000 resolution with a target AGC of 2 × 10^5^ ions and max injection time of 120 ms. An isolation width of 0.7 m/z and normalized collision energy of 28% were used.

A reference plasma sample from a pool of all EPL plasma samples from the verification cohort was depleted, digested, and spiked with SIL peptide standards as described above, and then aliquots of the final digest with added internal standard SIL peptides were snap frozen. An aliquot of this reference sample was typically run at the beginning, middle, and end of each set of samples to monitor variations in PRM signal intensities caused by changes in performance of the HPLC, reversed-phase column, or mass spectrometer.

### MS data analysis

Raw mass spectrometric data from the proteomics discovery were searched against the human UniProt database (released 8/29/16) and processed using label-free quantitation (LFQ) with MaxQuant (v. 1.5.2.8) [17], and the “match between runs” option [18] as previously described [19]. Protein identifications were filtered using Perseus software (v. 1.6.2.3; http://www.perseus-framework.org) [20] to remove decoy database reverse identifications, contaminants, proteins identified only by site modified peptides, or proteins identified by a single uniquely-mapping peptide.

In Perseus, protein group LFQ intensities were log2 transformed to reduce the impact of outliers. For pairwise comparisons of the discovery analysis, samples were categorized into groups based on pregnancy outcome (EP, IUP, or EPL). Protein groups having less than 50% of valid values (i.e., those with MS1 quantification results) present in every categorical group were removed. Prior to statistical analysis, missing data points were imputed from a Gaussian distribution of random numbers that simulate the distribution of low signal values (distribution width = 0.3, shift = 1.8). Perseus was also used for data visualization using volcano plots.

For PRM-MS analyses of the verification cohort, raw data files were analyzed using Skyline (v. 21.2) [21], and automated fragment ion selection (5 ions/peptide) was utilized. The summed peak area of the 3–4 most intense fragment ions was used to quantify both “light” (i.e., endogenous) and “heavy” (i.e., SIL) peptides. Missing peaks and/or peptide fragment peaks with mass error >10 ppm were removed. For peptides containing methionine, both the oxidized and non-oxidized forms were quantified separately, and peak areas were summed prior to calculating abundance.

Calibration curves of individual SIL peptides were prepared using an EPL plasma pool as a background to evaluate matrix effects. To determine linear ranges, upper limits of quantitation (ULOQ), and lower limits of quantitation (LLOQs), a seven-point dilution series of the SIL peptide pool (range: 0.64 fmol-10 pmol) was spiked into the EPL plasma pool and analyzed in duplicate by PRM-MS. Skyline was used to plot linear calibration curves and 1/x^2^ weighting was used. Peptides quantified in the 74 individual plasma samples that had quantities below the LLOQs were set to zero.

The abundance of each targeted peptide was calculated as the ratio between the light peptide and heavy peptide (L/H ratio). The amount of light peptide was calculated from the L/H ratio times the amount of heavy peptide spiked into the sample. Protein level in each sample was determined by taking the average of its targeted quantified peptides and the final protein concentration was calculated based on the volume of plasma analyzed.

### Statistical methods

Statistical analyses were performed using Perseus software (v.1.6.2.3), Microsoft Excel 2016, GraphPad Prism (v.5.04 and v.7), Stata 16, and R (v.4.2.1). For the discovery cohort, samples were grouped to identify differences related to early pregnancy complications such as EP or EPL. For the pairwise comparisons, two-tailed, two-sample Student’s t-test statistic was calculated, and a permutation-based false discovery rate (FDR) was applied (FDR ≤ 0.05, 250 permutations, S0 = 0.1) [22]. High priority (FDR ≤ 0.05) and additional significant (p ≤ 0.05 and fold change ≥ 3) candidate biomarkers were selected for further comparison between EP vs. non-EP (IUP + EPL) by a non-parametric Wilcoxon rank-sum test. Additionally, the area under the curve (AUC) from receiver operating characteristic (ROC) curves were assessed.

We aimed to have enough statistical power to verify the identified proteins from the discovery cohort in a verification cohort. Based on the data from the discovery cohort, we expected to verify markers with an effect size >0.7. Here, effect size refers to the difference between group means (EP vs. non-EP) divided by the pooled standard deviation. A verification set with 25 EP and 45 or more non-EP (IUP + EPL) would have at least 80% power at a two-sided type I error rate of 0.05 to verify a marker as long as its effect size is >0.7. To predict EP in the verification cohort, each biomarker was assessed using Wilcoxon rank-sum test with and without FDR adjustment calculated using Benjamini–Hochberg correction. Those markers with FDR ≤ 0.05 were further evaluated as potential predictors with least absolute shrinkage and selection operator (Lasso) regularization and logistic regression being used to explore biomarkers that may be used for better prediction of EP than models with a single predictor. For Lasso and logistical regression analyses, zero values were set as 0.01 and then the protein concentrations for all candidate biomarkers were log2 transformed. Correlations between candidate biomarkers were examined using Spearman’s rank correlation coefficient. Final predictors for the multivariable logistic model were selected using the Lasso technique with the 5-fold cross-validation and one-standard-error rule for determining the optimal tuning parameter. Due to the modest sample size of the study, variables selection was determined using 100 independent rounds runs of 5-fold cross-validation Lasso. The biomarkers that were selected 80 or more times from 100 runs were used as a final set of predictors in logistic model. Additionally, three other models were explored using protein substitutions based on the Spearman correlation cluster analysis. The predictive ability of the final logistic models was assessed by AUC, sensitivity, specificity, and accuracy, defined as the sum of true positives and true negatives divided by the total cohort size.

## Results

### Identification of candidate biomarkers in a discovery cohort

**Fig. 1:**
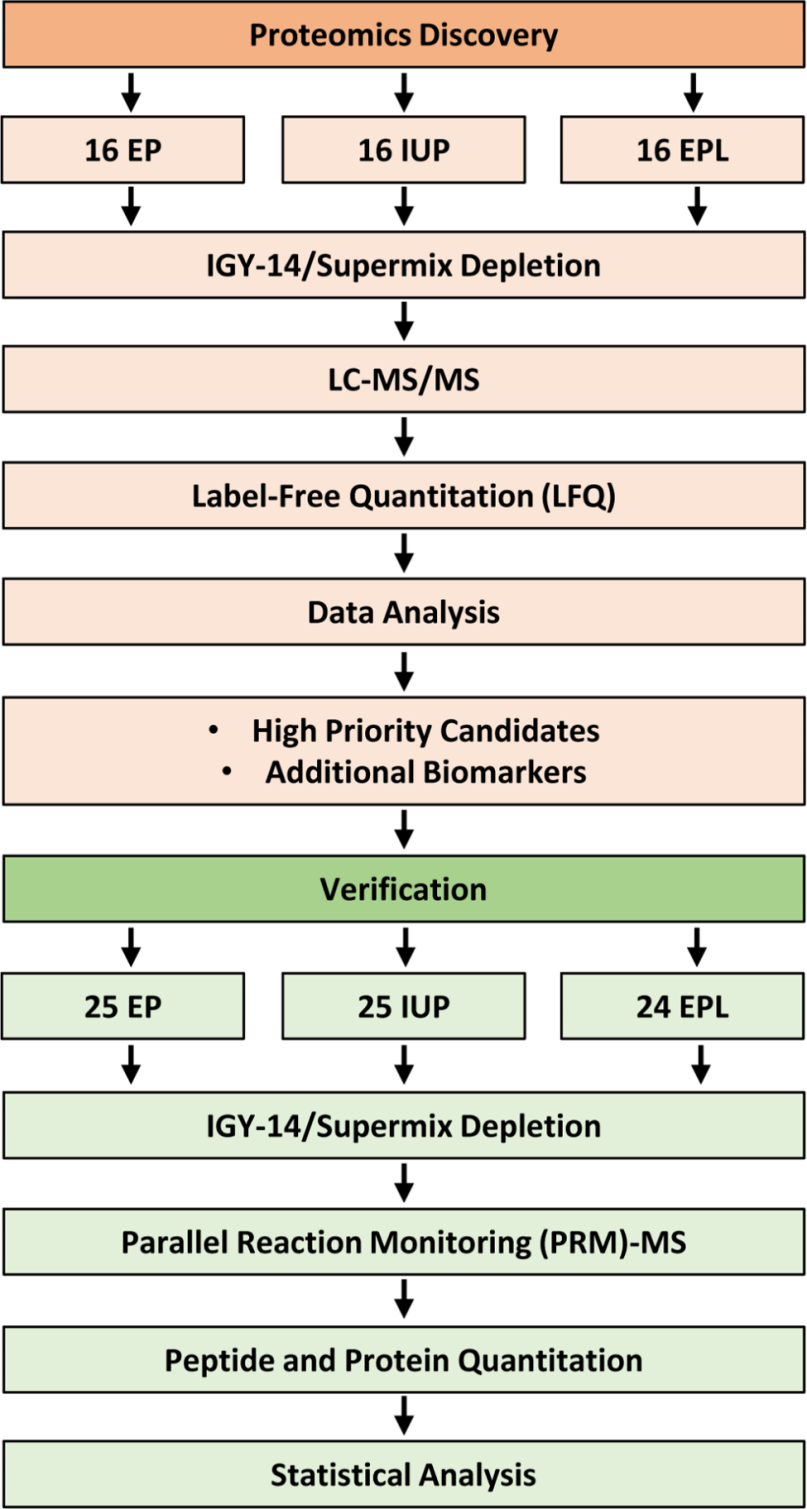
Scheme for discovery and verification of candidate biomarkers. Candidate biomarkers were identified by LC-MS/MS using label-free quantitation in a discovery cohort of 48 pregnant women. Biomarkers were then verified with targeted PRM-MS in an independent cohort of 74 women

**Fig. 2:**
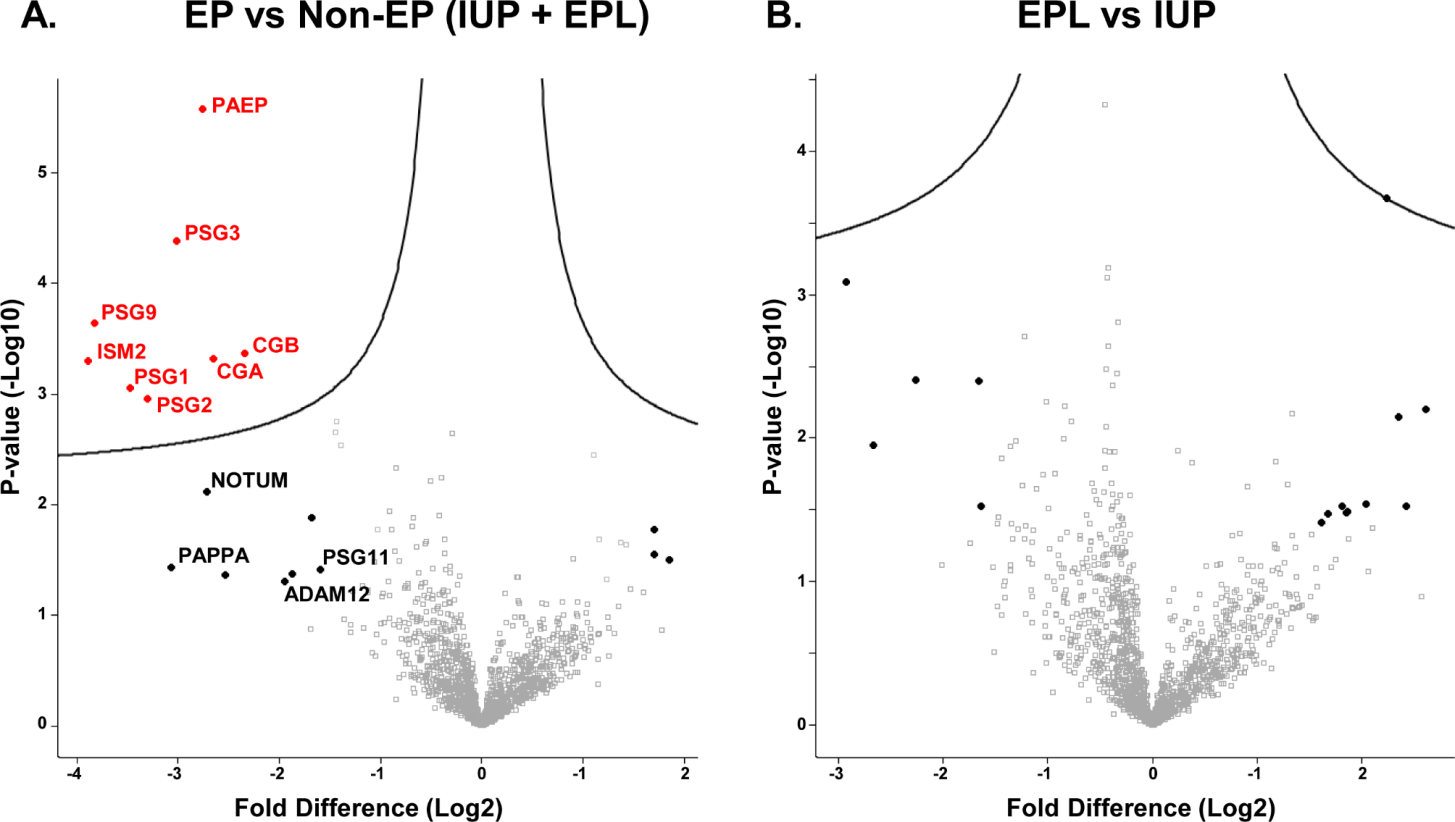
Identification of candidate biomarkers in the discovery cohort. (a) Volcano plot for EP vs. non-EP (IUP + EPL) (b) Volcano plot comparing EPL vs. IUP. High priority biomarkers outside the curves (FDR ≤ 0.05) are highlighted in red. Additional proteins having p ≤ 0.05 and fold change ≥ 3 are represented by black circles. Labeled proteins were further investigated in the verification cohort

**Fig. 3:**
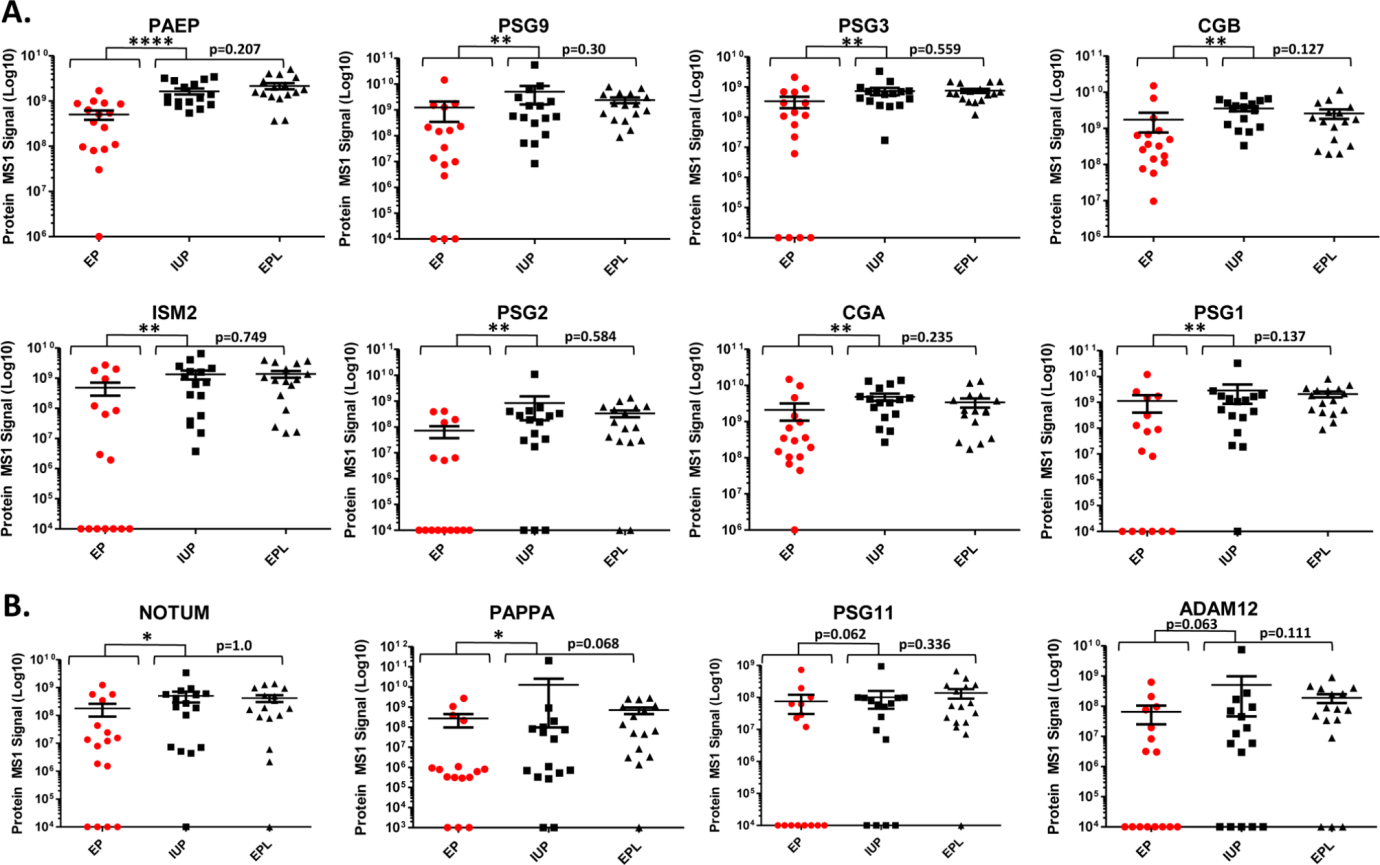
Scatterplots of candidate EP vs. non-EP biomarkers from the discovery cohort. Biomarkers were selected from volcano plots in Fig. 2a for further verification. (a) High-priority (FDR ≤ 0.05) (b) Additional candidate markers (p ≤ 0.05 and fold change ≥ 3). Wilcoxon rank sum test was used to compare EP vs. non-EP (IUP + EPL) and EPL vs. IUP. Statistical significance is shown above brackets (*<0.05, **<0.01, ***<0.001, not significant: p-value reported). For visualization, zero values are plotted on the x-axis

The scheme for label-free proteomics discovery of abundant-protein-depleted plasma samples from a cohort of 48 pregnant women with IUP, EP, and EPL is shown in Fig. 1. This analysis initially identified ~2200 proteins by 2 or more peptides. During preliminary data evaluation, we noticed a correlation between abundance levels of some proteins and collection date across clinical conditions. To avoid potential plasma storage bias, we systematically evaluated protein levels vs. total months the plasma was stored at -80 °C across all samples and filtered out proteins that showed decreased protein levels with increased length of storage (i.e., Pearson correlation with storage >-0.25). The remaining 1391 “storage stable” proteins were compared across clinical conditions. To identify potential biomarkers for the different clinical conditions, we performed all possible pairwise comparisons between the three groups as well as comparison of EP vs. non-EP (IUP + EPL). The most promising biomarkers were identified from the comparison of EP vs. non-EP, which yielded eight high priority candidates (FDR ≤ 0.05), including CGB, glycoprotein hormones alpha chain (CGA), isthmin-2 (ISM2), glycodelin (PAEP), pregnancy-specific beta-1-glycoprotein 1 (PSG1), pregnancy-specific beta-1-glycoprotein 2 (PSG2), pregnancy-specific beta-1-glycoprotein 3 (PSG3), and pregnancy-specific beta-1-glycoprotein 9 (PSG9) (Fig. 2a, Supplementary Table 1**)**. Interestingly, no high priority biomarkers were identified for EPL vs. IUP (Fig. 2b). In order to cast a relatively “wide net” for subsequent EP biomarker verification, we also considered several additional candidate biomarkers that did not pass the FDR cutoff but were significant based on Student’s t-test p-value ≤ 0.05 and fold change ≥ 3, including disintegrin and metalloproteinase domain-containing protein 12 (ADAM12), palmitoleoyl-protein carboxylesterase NOTUM (NOTUM), pappalysin-1 (PAPPA), and pregnancy-specific beta-1-glycoprotein 11 (PSG11) **(**Fig. 2a, Supplementary Table 1). Several other protein candidates meeting these relaxed criteria were also initially evaluated but subsequently dropped due to lack of reproducibly quantifiable, isoform-specific peptides as determined in preliminary PRM-MS assays. We also did not pursue several abundant blood proteins because their residual abundance levels were likely to be affected by variable recoveries from the major protein immunoaffinity depletion step. Scatter plots for the eight high priority and four additional significant candidate biomarkers across pregnancy outcomes are shown in Fig. 3. For each protein, the levels for EP samples were lower than for IUP and EPL samples. These results were consistent with our previous biomarker discovery analysis of serum pools from several EP and IUP patients where a general characteristic was that all candidate EP biomarkers were lower abundance in EP compared to IUP [23]. There was also extensive overlap between biomarkers identified in both discovery studies. Importantly, in the current, more in-depth proteome analysis with larger cohorts, the 12 candidate EP biomarkers exhibited good discriminatory capacity with AUC ≥ 0.664 for EP vs. non-EP (Table 2).

**Table 2:** Comparisons of AUC between candidate EP vs. non-EP biomarkers identified in the proteomics discovery cohort (N = 48)

Gene Name	Protein Description	AUC	Std. Error	95% Confidence Interval		P-value
PAEP	Glycodelin	0.898	0.046	0.808	0.989	< 0.0001
PSG9	Pregnancy-specific beta-1-glycoprotein 9	0.785	0.077	0.633	0.937	0.001
PSG3	Pregnancy-specific beta-1-glycoprotein 3	0.783	0.083	0.620	0.946	0.002
CGB	Choriogonadotropin subunit beta	0.779	0.083	0.617	0.942	0.002
ISM2	Isthmin-2	0.775	0.080	0.619	0.932	0.002
PSG2	Pregnancy-specific beta-1-glycoprotein 2	0.774	0.073	0.632	0.917	0.002
CGA	Glycoprotein hormones alpha chain	0.766	0.084	0.600	0.931	0.003
PSG1	Pregnancy-specific beta-1-glycoprotein 1	0.764	0.083	0.602	0.926	0.003
NOTUM	Protein notum homolog	0.703	0.084	0.539	0.867	0.023
PAPPA	Pappalysin-1	0.681	0.088	0.509	0.852	0.043
PSG11	Pregnancy-specific beta-1-glycoprotein 11	0.666	0.088	0.494	0.838	0.063
ADAM12	Disintegrin and metalloproteinase domain-containing protein 12	0.664	0.084	0.499	0.829	0.066

### Assessment of PRM-MS assay performance

We next developed a multiplexed quantitative PRM-MS assay that utilized SIL internal standard peptides for absolute quantitation of the 12 candidate EP biomarkers. An important feature in selecting targeted peptides was to ensure that the peptides could distinguish between highly homologous protein isoforms present in the blood including the very complex PSG protein family. We evaluated the robustness of the assay using multiple strategies based on the 36 monitored peptides selected for our candidate biomarkers (Supplementary Table 2). First, we determined the linearity, ULOQ, and LLOQ for each targeted peptide by producing a standard curve in a major protein depleted plasma background (Supplementary Table 2). The amounts of each peptide detected in each of the 74 individual plasma samples were compared to these limits, and values outside the range were flagged. All but one peptide, which was subsequently dropped, yielded quantifiable values for at least 90% of samples within at least one patient group.

We also prepared a reference plasma sample from a pool of EPL plasma samples (see Materials and Methods). Because the 74 individual plasma samples had to be processed and analyzed in multiple batches, this reference was used throughout the analyses to monitor consistency of quantitation both within an experiment and between batches by analyzing this sample at the beginning and end of each batch of LC-MS/MS runs when feasible. We observed excellent consistency of quantitation of the reference plasma sample across the entire experiment. The coefficients of variation (CVs) for most SIL peptides were < 25% within a series of LC-MS/MS runs and all peptides had CVs < 35% (Supplementary Table 2).

### Verification of EP versus non-EP candidate biomarkers in an independent cohort

**Fig. 4:**
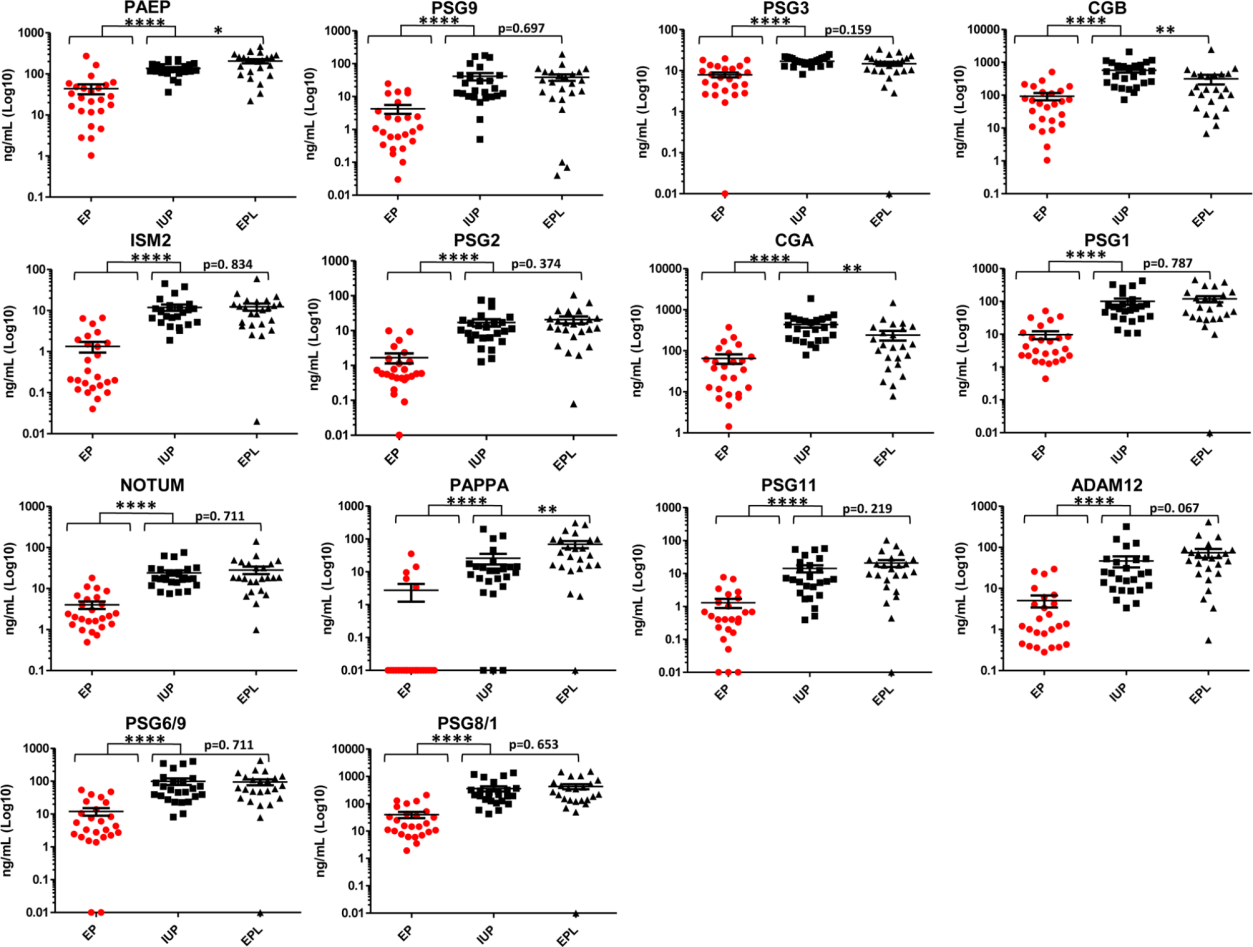
Scatterplots of candidate EP vs. non-EP biomarkers from the verification cohort. Wilcoxon rank sum test was used to compare EP vs. non-EP (IUP + EPL) and EPL vs. IUP. Statistical significance is shown above brackets (*<0.05, **<0.01, ***<0.001, not significant: p-value reported). For visualization, zero values are plotted on the x-axis

**Fig. 5:**
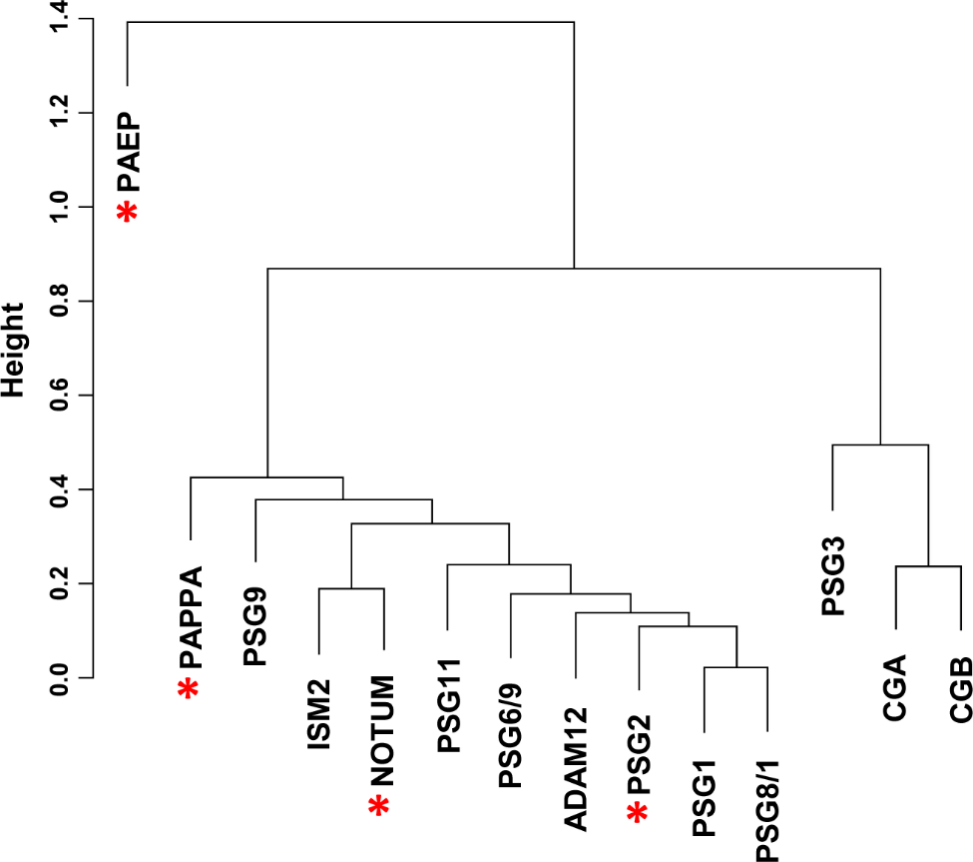
Correlation of predictors of EP vs. non-EP. Cluster dendrogram based on Spearman correlation. Lasso-selected protein markers (NOTUM, PAEP, PAPPA, and PSG2) are noted with red asterisks

**Fig. 6:**
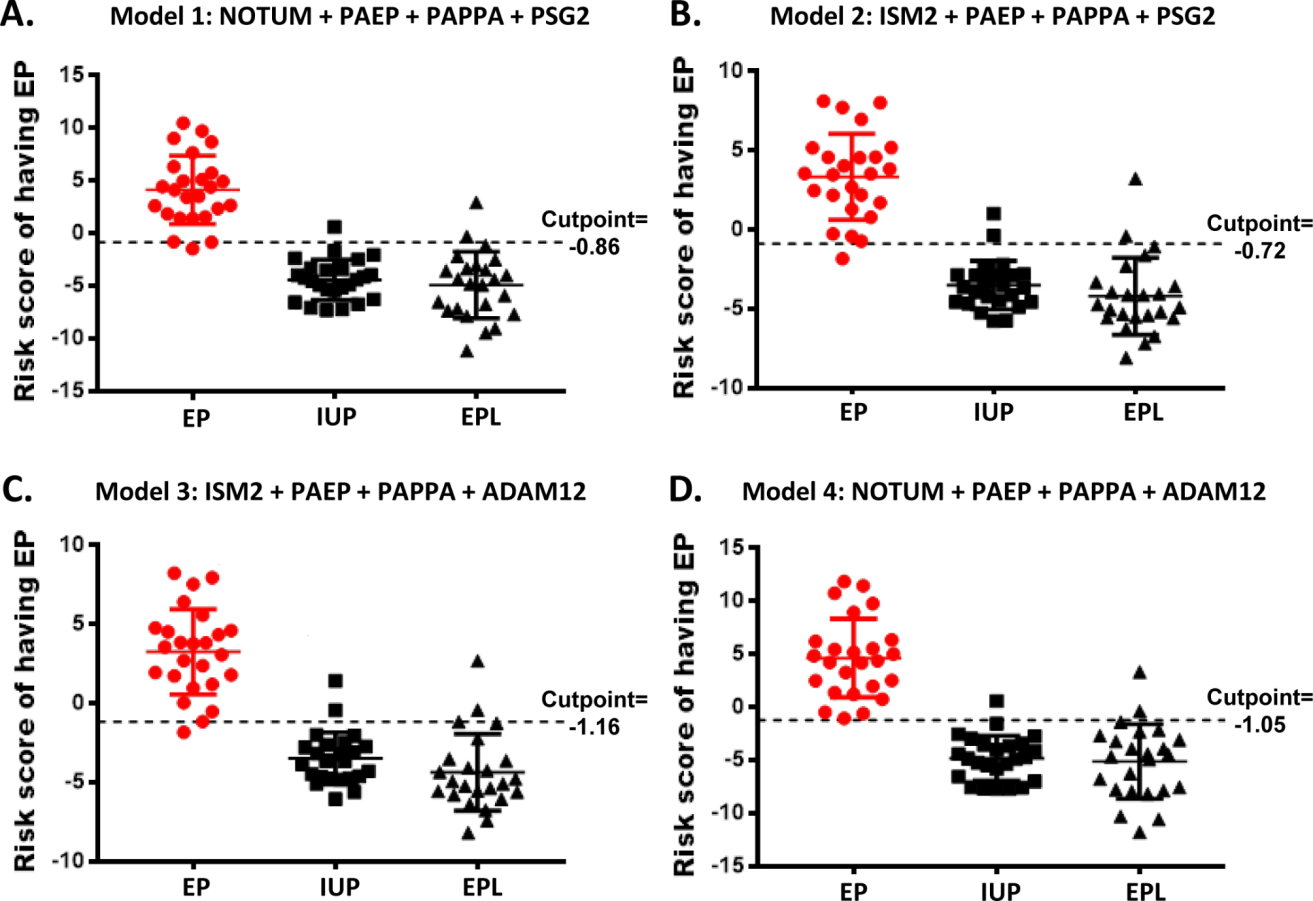
Prediction ability of multivariable logistics models for EP vs. non-EP. (a) Scatterplot showing the risk score of having EP by each group for the multivariable logistics model with predictors selected from Lasso (Model 1). (b) Prediction ability of Model 2 (using ISM2 instead of NOTUM). (c) Prediction ability of Model 3 (using ISM2 instead of NOTUM, and using ADAM12 instead of PSG2). (d) Prediction ability of Model 4 (using ADAM12 instead of PSG2)

We assessed the potential clinical utility of individual biomarkers by determining the ability of each biomarker to distinguish EP vs. non-EP in an independent cohort of 74 patients. Protein concentrations (ng/mL) for each of the markers in the 74 patient plasma samples are listed in Supplementary Table 3. We evaluated 12 unique protein markers (ADAM12, CGA, CGB, ISM2, NOTUM, PAEP, PAPPA, PSG1, PSG2, PSG3, PSG9, and PSG11) and two peptide markers that are shared between two pregnancy specific beta-1-glycoproteins (PSG6/9 and PSG8/1) and found that all markers were significantly different between EP and non-EP patients using Wilcoxon rank sum test (Fig. 4). Likewise, each of the individual biomarkers were excellent at accurately distinguishing EP vs. non-EP with AUCs ≥ 0.82. AUCs for all biomarkers were higher in the verification cohort than in the discovery cohort (Table 3). We further assessed the predictive ability of the 14 markers for EP using univariate logistic regression analysis (Supplementary Table 4) and multivariable logistic regression with Lasso-selected markers (NOTUM, PAEP, PAPPA, and PSG2) as predictors (Model 1 in Table 4). Spearman correlation coefficients indicated that some of the markers correlated with each other (Fig. 5, Supplementary Fig. 1). As expected, due to minimization of multicollinearity, the Lasso-selected protein markers were from different correlation clusters. We subsequently generated additional multivariable logistic regression models in which highly correlated protein markers, specifically NOTUM/ISM2 and PSG2/ADAM12, were exchanged to evaluate the effect on predictive ability for EP (Models 2–4 in Table 4). The multivariable logistic regression models were used to calculate risk scores for EP (Fig. 6a-d), and sensitivity, specificity, and accuracy were determined based on optimized cutpoints (Table 5). With a cutpoint of -0.858, the model using Lasso-selected predictors (Model 1) had a sensitivity of 96%, specificity of 93.9%, and accuracy of 94.6%. Model 4, in which PSG2 was exchanged for ADAM12, exhibited the best performance with a sensitivity of 100%, specificity of 93.9%, and accuracy of 96% at a risk score cutpoint of -1.05 (Table 5). Further, areas under the ROC curves were compared using an algorithm suggested by DeLong, DeLong, and Clarke-Pearson [24] (Table 6). The AUCs from the multivariable models were all higher than models with single predictors. The multivariable models had similar statistical significance by this measure; however, Model 4 again had the best performance with an AUC of 0.987 with 95% CI of 0.968 to 1.0.

**Table 3:** Comparisons of AUC between candidate EP vs. non-EP biomarkers identified in the verification cohort (N = 74)

Gene Name	AUC	Std. Error	95% Confidence Interval		P-value
ISM2	0.941	0.029	0.8851	0.9974	< 0.0001
NOTUM	0.939	0.029	0.8818	0.9957	< 0.0001
PSG8/1	0.936	0.030	0.8776	0.9934	< 0.0001
PSG2	0.935	0.029	0.8773	0.9929	< 0.0001
PSG1	0.931	0.031	0.8703	0.991	< 0.0001
ADAM12	0.916	0.034	0.8491	0.9819	< 0.0001
PAEP	0.915	0.042	0.833	0.9972	< 0.0001
PSG6/9	0.907	0.035	0.838	0.9758	< 0.0001
PSG11	0.897	0.036	0.826	0.9683	< 0.0001
PAPPA	0.894	0.040	0.8151	0.9727	< 0.0001
CGA	0.865	0.043	0.7806	0.9484	< 0.0001
PSG9	0.857	0.045	0.7692	0.9443	< 0.0001
PSG3	0.825	0.052	0.7229	0.9269	< 0.0001
CGB	0.820	0.049	0.7232	0.916	< 0.0001

**Table 4:** Multivariable logistic regression models for EP vs. non-EP (N = 74)

Predictors	Coefficient^a^	Std. Error	z	P>|z|	95% Confidence Interval	
Model 1: NOTUM + PAEP + PAPPA + PSG2						
NOTUM	-1.62	0.865	-1.87	0.061	-3.316	0.076
PAEP	-1.573	0.535	-2.94	0.003	-2.622	-0.524
PAPPA	-0.03	0.197	-0.15	0.878	-0.417	0.356
PSG2	0.097	0.52	0.19	0.853	-0.923	1.117
Constant	13.282	4.562	2.91	0.004	4.341	22.223
Model 2: ISM2 + PAEP + PAPPA + PSG2						
ISM2	-0.31	0.386	-0.8	0.422	-1.066	0.447
PAEP	-1.349	0.444	-3.04	0.002	-2.22	-0.478
PAPPA	-0.109	0.174	-0.63	0.531	-0.45	0.232
PSG2	-0.338	0.476	-0.71	0.477	-1.27	0.594
Constant	8.312	2.881	2.89	0.004	2.665	13.958
Model 3: ISM2 + PAEP + PAPPA + ADAM12						
ISM2	-0.336	0.421	-0.8	0.424	-1.161	0.488
PAEP	-1.349	0.443	-3.05	0.002	-2.216	-0.482
PAPPA	-0.141	0.163	-0.86	0.387	-0.462	0.179
ADAM12	-0.236	0.503	-0.47	0.639	-1.223	0.75
Constant	8.43	2.944	2.86	0.004	2.66	14.2
Model 4: NOTUM + PAEP + PAPPA + ADAM12						
NOTUM	-2.201	1.159	-1.9	0.058	-4.474	0.071
PAEP	-1.739	0.622	-2.8	0.005	-2.959	-0.52
PAPPA	-0.128	0.232	-0.55	0.58	-0.584	0.327
ADAM12	0.62	0.772	0.8	0.422	-0.892	2.133
Constant	14.289	5.071	2.82	0.005	4.351	24.227

^a^Coefficients and constants are used to calculate risk scores for each individual patient

**Table 5:** The sensitivity, specificity, and accuracy of each multivariable logistic model at optimal cutpoints (N = 74)

Predictors	Cutpoint	Sensitivity	Specificity	Accuracy
Model 1: NOTUM + PAEP + PAPPA + PSG2	≥ -0.858	96.00%	93.88%	94.59%
Model 2: ISM2 + PAEP + PAPPA + PSG2	≥ -0.722	96.00%	91.84%	93.24%
Model 3: ISM2 + PAEP + PAPPA + ADAM12	≥ -1.16	96.00%	91.84%	93.24%
Model 4: NOTUM + PAEP + PAPPA + ADAM12	≥ -1.05	100.00%	93.88%	95.95%

**Table 6:** Comparisons of AUC between each logistic model with single predictors versus each multivariable logistic model predicting EP vs. non-EP (N = 74)

Predictors	AUC	Std. Error	95% Confidence Interval		Model 1: P-value^a^	Model 2: P-value^a^	Model 3: P-value^a^	Model 4: P-value^a^
Model 1: NOTUM + PAEP + PAPPA + PSG2	0.986	0.010	0.967	1.000	Reference	N/A	N/A	N/A
Model 2: ISM2 + PAEP + PAPPA + PSG2	0.980	0.012	0.957	1.000	N/A	Reference	N/A	N/A
Model 3: ISM2 + PAEP + PAPPA + ADAM12	0.980	0.012	0.957	1.000	N/A	N/A	Reference	N/A
Model 4: NOTUM + PAEP + PAPPA + ADAM12	0.987	0.010	0.968	1.000	N/A	N/A	N/A	Reference
ISM2	0.941	0.029	0.885	0.997	0.051	0.071	0.078	0.046
NOTUM	0.939	0.029	0.882	0.996	0.049	0.073	0.078	0.044
PSG8/1	0.936	0.030	0.878	0.993	0.038	0.056	0.061	0.034
PSG2	0.935	0.030	0.877	0.993	0.031	0.035	0.039	0.027
PSG1	0.931	0.031	0.870	0.991	0.030	0.041	0.044	0.027
ADAM12	0.916	0.034	0.849	0.982	0.018	0.022	0.023	0.017
PAEP	0.915	0.042	0.832	0.998	0.081	0.106	0.104	0.081
PSG6/9	0.907	0.035	0.838	0.976	0.009	0.011	0.011	0.008
PSG11	0.897	0.036	0.826	0.968	0.004	0.004	0.005	0.004
PAPPA	0.894	0.039	0.817	0.970	0.012	0.013	0.013	0.011
CGA	0.865	0.043	0.780	0.949	0.003	0.004	0.004	0.003
PSG9	0.857	0.045	0.769	0.945	0.002	0.002	0.002	0.002
PSG3	0.825	0.053	0.722	0.928	0.001	0.002	0.002	0.001
CGB	0.820	0.049	0.723	0.916	0.000	0.001	0.001	0.000

^a^Single predictor models vs. multivariable model; N/A: Not applicable

## Discussion

In a proteomics discovery of plasma samples from women having IUP, EP, or EPL, we sought to discover biomarkers that could distinguish any of the three pregnancy outcomes from one or more of the other outcomes. We identified 12 promising biomarkers for EP vs. non-EP (IUP + EPL) but no high priority biomarkers that distinguish EPL from IUP (Fig. 2). Hence, for verification in an independent patient cohort using a different MS method, we focused on biomarkers of EP vs. non-EP including CGB, CGA, ADAM12, ISM2, NOTUM, PAEP, PAPPA PSG1, PSG2, PSG3, PSG9, PSG11, PSG6/9, and PSG8/1. Most of the markers that we describe, including CGB, CGA, PAPPA, ADAM12, ISM2, NOTUM, and the PSGs are known to be associated with trophoblast function [25–28]. PAEP is secreted from the endometrium and fallopian tube and has an immunomodulatory role in implantation [26]. Hence, it is reasonable that a fetus implanted outside of the uterus would have anomalous levels of these markers compared with normal pregnancy development. Additionally, we identified many of these proteins (ADAM12, ISM2, PAEP, PAPPA, PSG1, PSG2, CGB, and CGA) in a prior pilot discovery analysis of six small serum pools from women with EP and IUP [23]. Likewise, a follow-up study used a commercial dissociation-enhanced lanthanide fluoroimmunoassay (DELFIA) assay to validate ADAM12 in 199 serum specimens from women with EP and IUP [29]. However, that assay was limited by sensitivity because a substantial portion of patients had ADAM12 levels that fell below the detection limit. Other studies evaluating ADAM12 as a predictor of EP and other adverse pregnancy outcomes have had conflicting results. Yang et al. found that ADAM12 maintained low levels in EP and miscarriage compared to normal pregnancies [30], while Horne et al. determined that ADAM12 values were increased in EP compared to IUP or miscarriage and, that ADAM12 had limited value as a diagnostic marker for EP when measured in isolation [31]. However, because of the proprietary nature of most commercial antibody assays, the specific domain that was targeted by the antibody and potential cross-reactivity with isoforms and homologs in these studies was unknown.

A critical component of biomarker verification is the accurate, unambiguous quantitation of the target protein isoform while distinguishing homologous family members because an assay that simultaneously measures homologous proteins that do not correlate with the clinical condition will likely reduce accuracy of the diagnosis. This confounding effect is not surprising as related proteins are often associated with distinct structural or functional roles and will not *a priori* track together with a clinical condition [32, 33]. Assays with poorly defined isoform specificity, such as sandwich ELISA assays that lack rigorous antibody characterization, have the potential to yield misleading results if multiple related proteins are quantitated together.

The presence of homologous proteins in blood can also complicate protein quantitation using label-free LC-MS/MS analysis because it is not apparent how peptides shared between identified proteins should be distributed between homologs. Specifically, our discovery proteomics analysis of 48 plasma samples identified several candidate biomarkers with ambiguous assignment of shared peptides to highly homologous protein isoforms. This was a particular challenge for the PSG family of proteins. At least nine out of ten possible PSG gene products were detected in plasma, reflecting the abundant nature of these proteins in maternal blood during pregnancy [34, 35]. The extensive sharing of common peptides across isoforms and stochastic detection of many of these peptides limited the accuracy of quantitation of these proteins in the discovery cohort. To circumvent this complication in the targeted PRM-MS assay, we based protein quantitation on targeted tryptic peptides that were unique to putative diagnostic isoforms plus two shared peptides (see below). Because most single amino acid substitutions can be readily distinguished by MS, targeted MS-based quantitation methods such as multiple reaction monitoring (MRM) and PRM-MS has been used successfully to quantitate specific protein isoforms from cell extracts as well as biofluids [36–40]. Also, we previously showed that quantitative MRM-MS assays with high isoform specificity could be used to distinguish between all potential highly homologous protein isoforms in ovarian cancer patient sera [41].

For the PSGs, we targeted peptides that were unique to the putative diagnostic isoforms (PSG1, PSG2, PSG3, PSG9, and PSG11), as well as two peptides that were shared between PSG isoforms that were ambiguous from the discovery analysis (PSG1/8 and PSG 6/9). We did not find that these shared peptide markers provided a strong improvement for EP prediction compared to their unique counterparts from PSG1 and PSG9, respectively (Table 3). Therefore, we determined that there was no clear advantage of pursuing these shared markers and de-emphasized them when developing predictive models for EP. Further, it should be noted that, although the individual PSGs are each highly significant candidates, they may be difficult to validate by ELISA assays as part of a clinical biomarker panel unless the assay was rigorously demonstrated to quantify only a single isoform from among the nine isoforms present in patient plasma. In this regard, it should be emphasized that the other four PSG isoforms detected in the discovery study did not correlate with any of the clinical groups and would therefore confound accurate quantitation in assays without sufficient isoform specificity.

It is interesting that all 12 biomarkers from the discovery study were verified and had higher AUCs in the larger, independent verification cohort. We primarily attribute this higher performance as biomarkers to the superior quantitative performance of targeted PRM-MS assays compared with LFQ discovery proteomics where the accuracy of protein quantitation is somewhat limited by (1) variable detection and quantitation of specific peptides across samples, (2) potential inconsistent distribution of shared peptides to different isoforms, and (3) inaccurate distribution of shared peptides to the wrong isoform.

After assessing the predictive ability of the 14 candidate markers both individually and in combinations, we determined that the performance of multivariable logistic regression models was higher than those models with single predictors **(**Table 6**)**. We identified an initial multiple biomarker panel using Lasso feature selection (Model 1: NOTUM, PAEP, PAPPA, PSG2) that has the predictive capacity to identify an EP with high accuracy (94.6%, Table 5). We evaluated several alternative panels based on the Lasso-selected model in which highly correlated biomarkers were substituted (i.e., NOTUM with ISM2; PSG2 with ADAM12) and found that they perform similarly to one another (Table 5). The biomarker panel with the highest performance was Model 4 (NOTUM, PAEP, PAPPA, ADAM12) with an AUC of 0.987 and accuracy of 96%; however, because all four models performed similarly with regard to EP predictive ability, all candidate biomarkers listed in these models as well as other closely correlated biomarkers should be considered in future studies.

The current clinical marker for EP is CGB, and our discovery analysis found that it, along with its fellow chorionic gonadotropin alpha subunit CGA, was among the candidate proteins that distinguished EP from other pregnancy outcomes. However, neither CGB nor CGA were among the features selected by the Lasso analysis and incorporation of CGB, CGA, or the highly correlated marker PSG3 did not significantly improve performance of the models. Importantly, we note that all the other tested individual candidate biomarkers and multiprotein panels had higher AUCs than CGB alone (Table 6).

We also explored the minimum number of biomarkers needed for accurate diagnosis of EP by evaluating logistic regression models based on the Lasso-selected model with fewer features. We found that discrimination ability based on AUC was somewhat reduced when three or fewer markers were incorporated into a model. However, there is a tradeoff between the performance of a biomarker panel and the feasibility of clinical implementation and further verification studies on multiple patient populations may show that subsets of the four biomarker panels in one or more of the above models may be sufficient for highly accurate diagnosis of EP. There have been a number of prior studies evaluating combinations of markers for their effectiveness in diagnosing EP, including several of the candidate markers from this study. For example, a triple marker analysis of PAPPA plus progesterone and VEGF clearly discriminated EP in serum from 43 women with EP compared to 79 women with normal IUP, and the triple marker combination was superior to single marker measurements [42]. Further, in a larger study of serum from 230 women with EP, IUP, and EPL, PAEP and ADAM12 combined with Activin-A definitively classified pregnancy location (EP vs. IUP + EPL) in 29% of the samples with 100% accuracy for EP. Measurement of PAPPA plus progesterone was better at classifying viability (IUP vs. EP + EPL) in 61% of the samples with 94% accuracy in that study [1]. It should be noted that in addition to the use of ELISAs, the specific marker combinations and statistical methods for discriminating EP from other pregnancy outcomes were different from our targeted PRM-MS analysis.

The major limitation of this study is the moderate sample size of each pregnancy group being evaluated. It is important to avoid over-fitting the data when generating predictive models for patient risk from limited sample sets. Validation of larger cohorts will be necessary to demonstrate the clinical applicability of our biomarker panels, both in relation to the clinically used biomarker CGB and to distinguish between the similarly performing multi-protein biomarker panels. We recognize that MS analysis of proteins is not routinely used in clinical diagnostic laboratories, although its use is becoming more common and offers the advantage of unmatched protein isoform specificity. Our use of a multiplexed, targeted PRM-MS assay was primarily to unambiguously quantify related isoforms for robust biomarker verification. Future development of ELISA assays that accurately quantify the proteins in our multi-marker panels is the more conventional path toward developing a routine clinical assay. While it would likely be very difficult to develop truly isoform-specific assays for PSG family proteins, our best performing biomarker panel (NOTUM, PAEP, PAPPA, ADAM12) does not contain any of the PSG isoforms and therefore may be more amenable for ELISAs than panels that include one or more PSG isoforms. The major alternative to a multiple biomarker ELISA assay is to implement a higher-throughput targeted MS assay, and efforts to streamline the assay are being pursued.

## Conclusions

This study used discovery proteomics to identify 12 plasma protein biomarkers that distinguish EP from either IUP or EPL, and all 12 proteins were verified in a larger, independent patient cohort using a more quantitatively accurate, multiplexed PRM-MS assay. Highly accurate diagnosis of EP could be achieved using a four-protein biomarker panel consisting of NOTUM, PAEP, PAPPA, and ADAM12 with an algorithm combining these biomarkers to calculate an EP risk score having an AUC of 0.987 and accuracy of 96%. In addition, several other biomarkers closely correlate with three of these biomarkers and other models that substitute closely clustered biomarkers perform similarly. In addition to the four-protein models presented here, several other substitutions are feasible. As different combinations of biomarkers may perform somewhat differently in larger follow-up validation studies, these results suggest that further validation studies should focus on nine proteins (PAEP, PAPPA, PSG9, ISM2, NOTUM, PSG11, ADAM12, PSG2, and PSG1) using assays that accurately quantitate specific isoforms in the presence of highly homologous isoforms.

### Electronic supplementary material

Below is the link to the electronic supplementary material.

Supplementary Material 1

Supplementary Material 2

Supplementary Material 3

Supplementary Material 4

Supplementary Material 5

## Data Availability

The mass spectrometry proteomics data have been deposited to the MassIVE data repository with the accession number MSV000091300 and the ProteomeXchange Consortium [43] with the accession number PXD040189.

## Abbreviations

IUP: intrauterine pregnancy
EPL: early pregnancy loss
EP: ectopic pregnancy
β-hCG: β-human chorionic gonadotropin
CGB: Choriogonadotropin subunit beta
LC-MS/MS: liquid chromatography-tandem mass spectrometry
PRM: parallel reaction monitoring
ELISA: enzyme-linked immunosorbent assay
GA: gestational age
SIL: stable isotope label
Qtag: quantitation tag
UPLC: ultrahigh pressure liquid chromatography
RP-HPLC: reverse phase-high pressure liquid chromatography
LFQ: label-free quantitation
ULOQ: upper limit of quantitation
LLOQ: lower limit of quantitation
FDR: false discovery rate
AUC: area under the receiver operator curve
Lasso: least absolute shrinkage and selection operator
CGA: Glycoprotein hormones alpha chain
ISM2: Isthmin-2
PAEP: Glycodelin
PSG1: Pregnancy-specific beta-1-glycoprotein 1
PSG2: Pregnancy-specific beta-1-glycoprotein 2
PSG3: Pregnancy-specific beta-1-glycoprotein 3
PSG9: Pregnancy-specific beta-1-glycoprotein 9
ADAM12: Disintegrin and metalloproteinase domain-containing protein 12
NOTUM: Palmitoleoyl-protein carboxylesterase NOTUM
PAPPA: Pappalysin-1
PSG11: Pregnancy-specific beta-1-glycoprotein 11
CV: Coefficient of variation
MRM: multiple reaction monitoring
DELFIA: dissociation-enhanced lanthanide fluoroimmunoassay

## References

[1] Senapati S, Sammel MD, Butts SF, Takacs P, Chung K, Barnhart KT (2016). Predicting first trimester pregnancy outcome: derivation of a multiple marker test. Fertil Steril.

[2] Barnhart KT (2009). Clinical practice. Ectopic pregnancy. N Engl J Med.

[3] Ammon Avalos L, Galindo C, Li DK (2012). A systematic review to calculate background miscarriage rates using life table analysis. Birth Defects Res a Clin Mol Teratol.

[4] Jurkovic D, Overton C, Bender-Atik R (2013). Diagnosis and management of first trimester miscarriage. BMJ.

[5] American College of O, Gynecologists (2008). ACOG Practice Bulletin No. 94: medical management of ectopic pregnancy. Obstet Gynecol.

[6] Barnhart KT, Guo W, Cary MS, Morse CB, Chung K, Takacs P (2016). Differences in serum human chorionic gonadotropin rise in early pregnancy by race and value at Presentation. Obstet Gynecol.

[7] Bollig KJ, Senapati S, Sammel MD, Takacs P, Robins JC, Haisenleder DJ et al. Validation of a multiple marker test for early pregnancy outcome prediction. J Assist Reprod Genet. 2023. 10.1007/s10815-023-02719-w PMC10224881 36708430

[8] Wang H, Tang HY, Tan GC, Speicher DW (2011). Data analysis strategy for maximizing high-confidence protein identifications in complex proteomes such as human tumor secretomes and human serum. J Proteome Res.

[9] Gerszten RE, Asnani A, Carr SA (2011). Status and prospects for discovery and verification of new biomarkers of cardiovascular disease by proteomics. Circ Res.

[10] Gerszten RE, Carr SA, Sabatine M (2010). Integration of proteomic-based tools for improved biomarkers of myocardial injury. Clin Chem.

[11] Manglani M, Rua R, Hendricksen A, Braunschweig D, Gao Q, Tan W (2019). Method to quantify cytokines and chemokines in mouse brain tissue using Bio-Plex multiplex immunoassays. Methods.

[12] Song J, Merbs SL, Sokoll LJ, Chan DW, Zhang Z (2019). A multiplex immunoassay of serum biomarkers for the detection of uveal melanoma. Clin Proteom.

[13] Barnhart K, van Mello NM, Bourne T, Kirk E, Van Calster B, Bottomley C (2011). Pregnancy of unknown location: a consensus statement of nomenclature, definitions, and outcome. Fertil Steril.

[14] Beer LA, Ky B, Barnhart KT, Speicher DW (2017). In-Depth, reproducible analysis of human plasma using IgY 14 and SuperMix Immunodepletion. Methods Mol Biol.

[15] Goldman AR, Beer LA, Tang HY, Hembach P, Zayas-Bazan D, Speicher DW. Proteome Analysis using Gel-LC-MS/MS. Curr Protoc Protein Sci. 2019:e93. 10.1002/cpps.93 PMC6653605 31180188

[16] Schnatbaum K, Zerweck J, Nehmer J, Wenschuh H, Schutkowski M, Reimer U. SpikeTides™—proteotypic peptides for large-scale MS-based proteomics. Nat Methods. 2011;8.

[17] Cox J, Mann M (2008). MaxQuant enables high peptide identification rates, individualized p.p.b.-range mass accuracies and proteome-wide protein quantification. Nat Biotechnol.

[18] Geiger T, Wehner A, Schaab C, Cox J, Mann M (2012). Comparative proteomic analysis of eleven common cell lines reveals ubiquitous but varying expression of most proteins. Mol Cell Proteomics.

[19] Beer LA, Liu P, Ky B, Barnhart KT, Speicher DW (2017). Efficient quantitative comparisons of plasma proteomes using label-free analysis with MaxQuant. Methods Mol Biol.

[20] Tyanova S, Temu T, Sinitcyn P, Carlson A, Hein MY, Geiger T (2016). The Perseus computational platform for comprehensive analysis of (prote)omics data. Nat Methods.

[21] Pino LK, Searle BC, Bollinger JG, Nunn B, MacLean B, MacCoss MJ (2020). The Skyline ecosystem: Informatics for quantitative mass spectrometry proteomics. Mass Spectrom Rev.

[22] Tusher VG, Tibshirani R, Chu G (2001). Significance analysis of microarrays applied to the ionizing radiation response. Proc Natl Acad Sci U S A.

[23] Beer LA, Tang HY, Sriswasdi S, Barnhart KT, Speicher DW (2011). Systematic discovery of ectopic pregnancy serum biomarkers using 3-D protein profiling coupled with label-free quantitation. J Proteome Res.

[24] DeLong ER, DeLong DM, Clarke-Pearson DL (1988). Comparing the areas under two or more correlated receiver operating characteristic curves: a nonparametric approach. Biometrics.

[25] Jeyarajah MJ, Jaju Bhattad G, Kelly RD, Baines KJ, Jaremek A, Yang FP (2022). The multifaceted role of GCM1 during trophoblast differentiation in the human placenta. Proc Natl Acad Sci U S A.

[26] Soundravally R, Pooja D. Biomarkers of Ectopic Pregnancy-Present and Future. 2015.

[27] Martinez C, Gonzalez-Ramirez J, Marin ME, Martinez-Coronilla G, Meza-Reyna VI, Mora R (2020). Isthmin 2 is decreased in preeclampsia and highly expressed in choriocarcinoma. Heliyon.

[28] Marchand M, Horcajadas JA, Esteban FJ, McElroy SL, Fisher SJ, Giudice LC (2011). Transcriptomic signature of trophoblast differentiation in a human embryonic stem cell model. Biol Reprod.

[29] Rausch ME, Beer L, Sammel MD, Takacs P, Chung K, Shaunik A (2011). A disintegrin and metalloprotease protein-12 as a novel marker for the diagnosis of ectopic pregnancy. Fertil Steril.

[30] Yang J, Wu J, Guo F, Wang D, Chen K, Li J (2014). Maternal serum disintegrin and metalloprotease protein-12 in early pregnancy as a potential marker of adverse pregnancy outcomes. PLoS ONE.

[31] Horne AW, Brown JK, Tong S, Kaitu’u-Lino T (2012). Evaluation of ADAM-12 as a diagnostic biomarker of ectopic pregnancy in women with a pregnancy of unknown location. PLoS ONE.

[32] Garcia-Blanco MA, Baraniak AP, Lasda EL (2004). Alternative splicing in disease and therapy. Nat Biotechnol.

[33] Kriventseva EV, Koch I, Apweiler R, Vingron M, Bork P, Gelfand MS (2003). Increase of functional diversity by alternative splicing. Trends Genet.

[34] Moore T, Dveksler GS (2014). Pregnancy-specific glycoproteins: complex gene families regulating maternal-fetal interactions. Int J Dev Biol.

[35] Moore T, Williams JM, Becerra-Rodriguez MA, Dunne M, Kammerer R, Dveksler G (2022). Pregnancy-specific glycoproteins: evolution, expression, functions and disease associations. Reproduction.

[36] Wang Q, Chaerkady R, Wu J, Hwang HJ, Papadopoulos N, Kopelovich L (2011). Mutant proteins as cancer-specific biomarkers. Proc Natl Acad Sci U S A.

[37] Zulak KG, Lippert DN, Kuzyk MA, Domanski D, Chou T, Borchers CH (2009). Targeted proteomics using selected reaction monitoring reveals the induction of specific terpene synthases in a multi-level study of methyl jasmonate-treated Norway spruce (Picea abies). Plant J.

[38] Andersson A, Remnestal J, Nellgard B, Vunk H, Kotol D, Edfors F (2019). Development of parallel reaction monitoring assays for cerebrospinal fluid proteins associated with Alzheimer’s disease. Clin Chim Acta.

[39] Kennedy JJ, Whiteaker JR, Ivey RG, Burian A, Chowdhury S, Tsai CF (2022). Internal standard triggered-parallel reaction monitoring Mass Spectrometry enables multiplexed quantification of candidate biomarkers in plasma. Anal Chem.

[40] Qin W, Qin X, Li L, Gao Y (2022). Proteome Analysis of urinary biomarkers in a bovine IRBP-Induced Uveitis Rat Model via Data-Independent Acquisition and parallel reaction monitoring proteomics. Front Mol Biosci.

[41] Tang HY, Beer LA, Tanyi JL, Zhang R, Liu Q, Speicher DW (2013). Protein isoform-specific validation defines multiple chloride intracellular channel and tropomyosin isoforms as serological biomarkers of ovarian cancer. J Proteom.

[42] Mueller MD, Raio L, Spoerri S, Ghezzi F, Dreher E, Bersinger NA (2004). Novel placental and nonplacental serum markers in ectopic versus normal intrauterine pregnancy. Fertil Steril.

[43] Vizcaino JA, Deutsch EW, Wang R, Csordas A, Reisinger F, Rios D (2014). ProteomeXchange provides globally coordinated proteomics data submission and dissemination. Nat Biotechnol.

