# Supplementary material for: Identification and verification of plasma protein biomarkers that accurately identify an ectopic pregnancy
Source: Clin Proteomics. 2023 Sep 15;20:37. doi: 10.1186/s12014-023-09425-w (PMC10503165; doi:10.1186/s12014-023-09425-w)
Supplement: Supplementary file 4 — Supplementary Material 4 [file 12014_2023_9425_MOESM4_ESM.docx]

**Supplementary Table 4.** Univariate logistic regression analysis results for EP vs. non-EP (N=74)

| **Predictors** | **Coefficient** | **Std. Error** | **z** | **P>\|z\|** | **95% Confidence Interval** | |
| --- | --- | --- | --- | --- | --- | --- |
| ADAM12 | -0.901 | 0.202 | -4.47 | <0.001 | -1.297 | -0.506 |
| CGA | -0.775 | 0.183 | -4.23 | <0.001 | -1.135 | -0.416 |
| CGB | -0.632 | 0.164 | -3.86 | <0.001 | -0.952 | -0.311 |
| ISM2 | -0.953 | 0.228 | -4.18 | <0.001 | -1.399 | -0.506 |
| NOTUM | -1.567 | 0.36 | -4.35 | <0.001 | -2.273 | -0.861 |
| PAEP | -1.588 | 0.367 | -4.33 | <0.001 | -2.307 | -0.869 |
| PAPPA | -0.362 | 0.07 | -5.16 | <0.001 | -0.499 | -0.224 |
| PSG1 | -0.799 | 0.183 | -4.36 | <0.001 | -1.158 | -0.44 |
| PSG11 | -0.648 | 0.155 | -4.19 | <0.001 | -0.951 | -0.345 |
| PSG2 | -0.991 | 0.216 | -4.58 | <0.001 | -1.415 | -0.567 |
| PSG3 | -0.589 | 0.257 | -2.29 | 0.022 | -1.093 | -0.086 |
| PSG6/9 | -0.707 | 0.174 | -4.05 | <0.001 | -1.048 | -0.365 |
| PSG8/1 | -0.756 | 0.179 | -4.22 | <0.001 | -1.107 | -0.404 |
| PSG9 | -0.473 | 0.115 | -4.11 | <0.001 | -0.699 | -0.248 |
