# Supplementary material for: Identification and verification of plasma protein biomarkers that accurately identify an ectopic pregnancy
Source: Clin Proteomics. 2023 Sep 15;20:37. doi: 10.1186/s12014-023-09425-w (PMC10503165; doi:10.1186/s12014-023-09425-w)
Supplement: Supplementary file 5 — Supplementary Material 5 [file 12014_2023_9425_MOESM5_ESM.pdf]

A

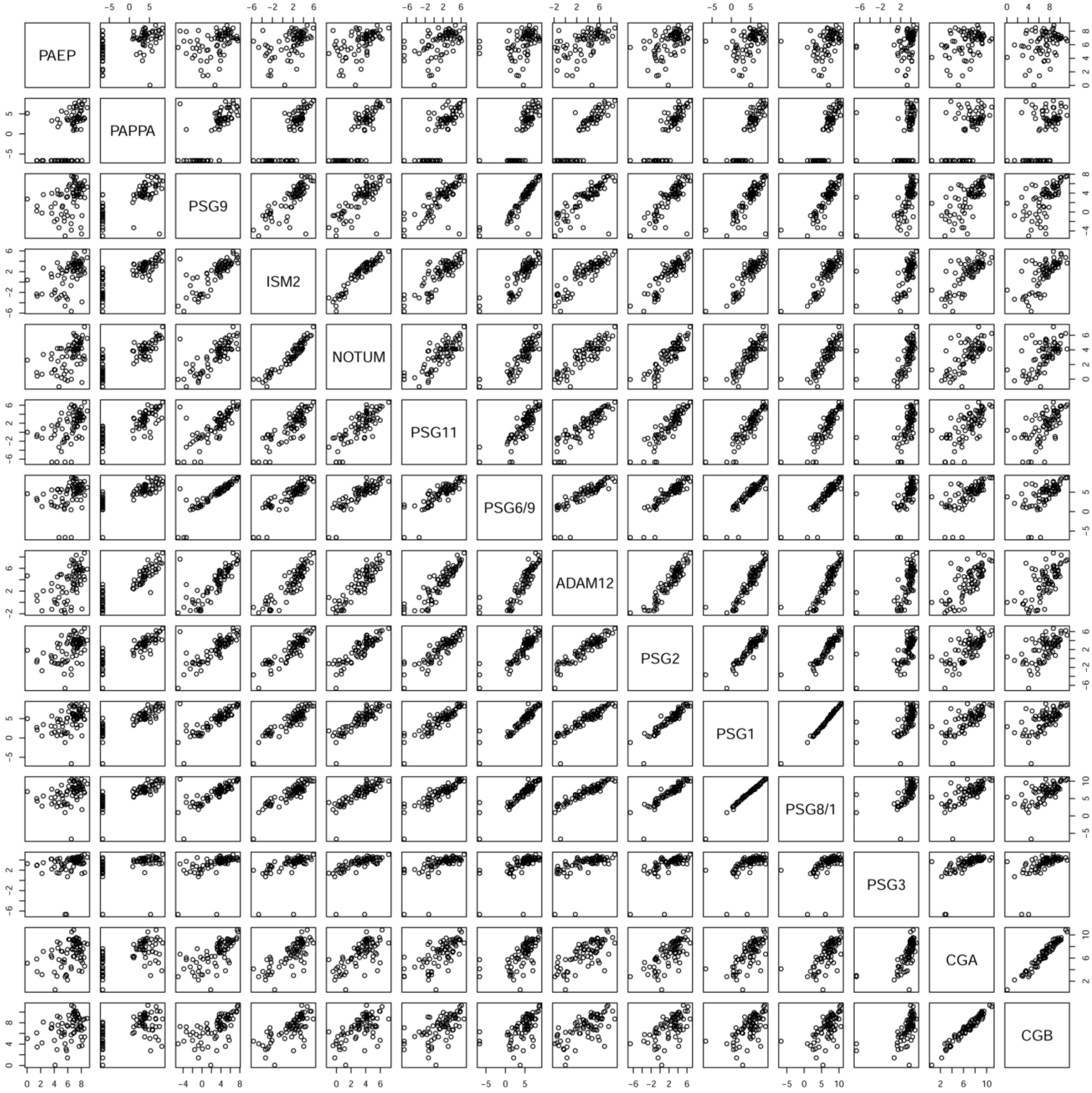

B

	PAEP	PAPPA	PSG9	ISM2	NOTUM	PSG11	PSG6/9	ADAM12	PSG2	PSG1	PSG8/1	PSG3	CGA	CGB
**PAEP**	1													
**PAPPA**	0.562	1												
**PSG9**	0.415	0.761	1											
**ISM2**	0.543	0.821	0.803	1										
**NOTUM**	0.544	0.825	0.752	0.940	1									
**PSG11**	0.547	0.807	0.794	0.788	0.768	1								
**PSG6/9**	0.478	0.836	0.932	0.856	0.843	0.872	1							
**ADAM12**	0.570	0.907	0.838	0.875	0.853	0.909	0.915	1						
**PSG2**	0.509	0.873	0.811	0.867	0.868	0.891	0.903	0.920	1					
**PSG1**	0.512	0.859	0.835	0.859	0.867	0.893	0.938	0.934	0.939	1				
**PSG8/1**	0.510	0.868	0.829	0.861	0.873	0.904	0.935	0.938	0.945	0.995	1			
**PSG3**	0.397	0.570	0.696	0.714	0.733	0.672	0.716	0.696	0.686	0.697	0.708	1		
**CGA**	0.295	0.521	0.735	0.725	0.642	0.698	0.729	0.698	0.707	0.728	0.728	0.771	1	
**CGB**	0.260	0.448	0.683	0.701	0.568	0.637	0.656	0.640	0.618	0.644	0.639	0.702	0.968	1

Supplementary Fig. 1 Spearman correlation of EP vs. non-EP candidate biomarkers from the verification cohort (N=74). a. Scatterplots for each candidate biomarker pair. b. Spearman correlation coefficients (r) for each candidate biomarker pair. Spearman rank correlation p ≤ 0.0005 for all protein pairs.
